# Are Barriers the Same Whether I Want to Start or Maintain Exercise? A Narrative Review on Healthy Older Adults

**DOI:** 10.3390/ijerph17176247

**Published:** 2020-08-27

**Authors:** Nathalie André, Nounagnon Frutueux Agbangla

**Affiliations:** 1 Centre de Recherches sur la Cognition et l’Apprentissage (UMR CNRS 7295), Université de Poitiers, 86000 Poitiers, France; 2 Maison des Sciences de l’Homme et de la Société, USR CNRS 3565, Université de Poitiers, 86000 Poitiers, France; 3 Université de Paris, EA 3625-Institut des Sciences du Sport-Santé de Paris (I3SP), 75015 Paris, France; 4 Unité de Recherche Pluridisciplinaire Sport Santé Société (URePSSS), ULR 7369, Univ. Artois, Univ. Lille, Univ. Littoral Côte d’Opale, F-59000 Lille, France

**Keywords:** barriers, older adults, exercise behavior, stages of change

## Abstract

To help older adults begin or adhere to regular physical exercise, several studies have endeavored to identify barriers to active behavior. However, there is a lack of information about barriers for active older people. In addition, most of the reviews of the literature compare only active people to inactive or sedentary people without examining in detail the barriers with respect to the degree of commitment to behavioral change. Finally, there is no consistency in the results of studies investigating the effects of barriers on the relationship between stages of change and exercise behavior. The first aim of this narrative review is to compare barriers that affect exercise stages of change from those that affect levels of exercise behavior in a healthy older population and the factors that can lead to relapse or dropout; the second aim is to identify the extent to which barriers hinder the relationships between stages of change and exercise behaviors. The results showed that barriers are well identified in sedentary people and in the first two stages of change (pre-contemplation and contemplation) compared to active seniors and other stages of change (preparation, action and maintenance). Consistency between the formulations of the different stages in comparison with the transtheoretical model and the definition of barriers and the limitations of measuring physical activity in the different studies are discussed. Finally, novel perspectives of research are proposed to address the flaws in the reviewed studies.

## 1. Introduction

The World Health Organization recommends that more attention be paid to increasing levels of physical activity [1]. These recommendations are based on a large number of epidemiological, cross-sectional and interventional studies that present a body of convincing evidence for the beneficial effects of regular practice of physical activity on seniors’ physical and cognitive health and social participation e.g., [2,3]. However, the odds of people being sufficiently active continue to decrease with increasing age [4]. According to Lee and colleagues, there are challenges to helping sedentary older people to start or adhere to regular exercise [5]. Older people may assume that health-promotion messages about exercise are aimed at younger adults or that their limitations in physical functioning prevent them from undertaking exercise. However, adherence to health-prevention programs is considered essential to achieving successful outcomes in physical exercise, including enhanced quality of life. Regular exercise and, more specifically, moderately intense physical activity have been recognized as some of the most significant health interventions for older adults [6,7,8]. With sedentary older people, the identification of reliable predictors allows healthcare providers to effectively structure interventions to promote changes in patterns of physical activity. Several reviews of the literature examining qualitative and quantitative studies on barriers and physical activity have identified conditions such as lack of time, lack of safe place and weather, environmental factors, physical problems and having no companions as mainly reported by sedentary older people [9,10,11,12,13,14,15]. In contrast, there is a lack of information concerning the barriers for exercisers; attrition most commonly occurs within 6 months of exercise initiation, with approximately 50% of participants dropping out before realizing any health benefits [16].

Researchers have used numerous theoretical models to understand the determinants of physical exercise [17,18,19]. Among them, the transtheoretical model (TTM) [20,21] is mainly used to determine how people change their behavior. This theoretical model is significant in that it focuses both on actual behavior and on behavioral intention. According to the TTM, behavior change is a dynamic process that occurs through a series of five interrelated stages: precontemplation, contemplation, preparation, action and maintenance [22,23]. It is well documented that exercise-related stages of change are related to levels of physical activity, self-efficacy, perceived benefits and costs for physical activity and processes of change [24]. However, some studies have examined to what extent barriers (perceived or actual) hinder the relationships between stages of change and exercise behavior [25,26,27], and the results have shown a lack of consistency. In addition, reviews examining barriers to physical activity among older adults generally examined age, gender, or type of physical activity [14,28,29]. No review has examined these barriers with regard to the energy expenditure in physical activity, and few studies have taken into account the stages of change by focusing on the role of the TTM in promoting physical activity [30,31,32]. Taking into account these two research domains (exercise psychology and health psychology) is relevant insofar as it helps to cross subjective and objective measurements of engagement in physical activity in older adults and to deepen the understanding of the links between the stages of change and the level of engagement in physical activity in terms of energy expenditure. Consequently, to examine the barriers during the different stages of behavior change among healthy older people, this review has two aims: first, to compare barriers that affect stages of change and those that affect levels of exercise behavior in a healthy older population and the factors that can lead to relapse or dropout; second, to identify to what extent barriers hinder the relationships between stages of change and exercise behaviors. Three questions guided the review process in each published report: (1) What barriers are reported by sedentary compared to active older adults? (2) What barriers are reported by older adults in the pre-action stage (i.e., precontemplation, contemplation and preparation) compared to the post-action stage (action and maintenance)? (3) To what extent do barriers predict exercise behavior change in older healthy people?

## 2. Literature Search

In order to write our narrative review, the PubMed, Web of Science and PsycINFO databases were searched (last search on June 2020) using the following keywords: (barrier) combined with (elderly OR older OR aging OR senior) AND (“physical activity” OR exercise) AND (“stages of change” OR “readiness of change”). Following this search, duplicate references have been removed. Then, the titles and abstracts of the retrieved papers were screened for relevance. Studies were included if they were written in English and if they reported barriers to physical activity in subjects aged >50 years old. When the sample included a larger age range, the article was included if data concerning the target ages were available. Papers were excluded if they examined palliative, frail or cognitively impaired patients. Studies aiming primarily to identify barriers to physical activity in older adults were excluded because several reviews of the literature already examined these studies [9,10,11,12,13,14,28,29].

## 3. Definitions of Key Concepts

### 3.1. Perceived Barriers

Perceived barriers belong to the decisional balance concept, which is a measure of attitude that captures how individuals weigh the consequences of a specific behavior in terms of pros and cons [33,34]. Applied to exercise behavior, decisional balance concerns the favorable and unfavorable consequences of taking up exercise as a lifestyle. In line with the TTM [21], perceived barriers, and also named perceived costs, are defined as one’s belief in the tangible and/or psychological costs of the advised behavior or as barriers that slow or halt completion of an ongoing health behavior.

### 3.2. Stages of Change

According to Marcus and Forsyth, people in precontemplation are not engaged in exercise and are not thinking about starting an exercise program [35]. Those in the contemplation stage are considering starting an exercise program. During the preparation stage, people participate in some physical activity but not at the level meeting Centers for Disease Control and American College of Sports Medicine (CDC/ACSM) guidelines [36]. People in the action stage have adopted regular exercise within the past 6 months but are at greater risk of not adhering than people in the maintenance stage, for whom exercise behavior has been established for 6 months or more.

### 3.3. Physical Activity

As already reported, the terms physical activity and exercise are frequently used interchangeably. Exercise is defined as a subtype of physical activity, specifically, “planned, structured, repetitive, and purposive bodily movement done to improve or maintain one or more components of physical fitness” [37] or as “a regular and structured subset of physical activity, performed deliberately and with a specific purpose such as the improvement of some aspect of health” [38]. Physical activity has been defined accordingly as “any bodily movements produced by skeletal muscles that result in energy expenditure”. In line with epidemiological studies [39,40], physical activity level has been divided into three categories: sedentary, defined as no sports or exercise reported in the past 2 weeks or no increase in heart rate reported from any activities; underactive, defined as not meeting the criteria for either the sedentary or the active category; and active, defined as either three or more sessions per week, for at least 20 min per session, of jogging-running, hiking, biking, swimming or dance resulting in a medium to large increase in reported heart rate or five or more sessions per week, for at least 30 min per session, of any physical activities (including walking, gardening or yard work, calisthenics, etc.) that resulted in at least some increase in reported heart rate.

## 4. Results

Our results highlight two main categories of studies. The first category concerns studies that have explored the relationship between barriers and exercise behavior. The second category includes studies that have explored the relationship between barriers and stages of change. Only studies with tangible results concerning energy expenditure, stages of change and barriers have been reported in the tables.

### 4.1. Relationship of Barriers with Exercise Behavior (N = 21)

Two questions are asked in this section. First, how are perceived barriers and physical activity and exercise measured? Second, what barriers are reported by sedentary and active people? By examining perceived barriers questionnaires, three formulations can be identified: (1) measures that assess barriers that prevent older adults from exercising [41,42]; (2) measures that assess reasons for not doing more physical activity [43,44] and (3) general measures assessing the degree to which barriers limit participation [45,46]. None of the previous studies clearly define the concept of perceived barriers. Nonetheless, formulations of items related to perceived barriers lead to some suggestions. For instance, in social cognitive theory (SCT), perceived barriers are defined as barriers that prevent individuals from initiating a health behavior such as exercising. Here, barriers provide information about the potential constraints on action as perceived by individuals. In line with this theoretical model, direct relations can be observed between perceived barriers and physical activity [47]. Regarding the ecological model, barriers are defined as real or perceived individual, interpersonal and contextual factors that prevent individuals from engaging in an activity or hinder their ability to do so [19,48]. This definition tends to weaken the study of the relationships between barriers and the level of physical activity for two main reasons. First, it becomes difficult to generalize results, and second, these definitions are less adapted to active people. On the other hand, much of the research measuring relationships between leisure time physical activity (LTPA) and perceived barriers has relied on retrospective questionnaires, which ask respondents to recall the frequency and duration of LTPA over an extended time period ranging from one week to several years. Such recall and summarizing of behavior are often inaccurate and are susceptible to bias, which could take the form of under- or over-reporting LTPA [49,50,51,52]. These biases might, thus, systematically affect estimates of relationships between LTPA and perceived barriers. Finally, more than 10 different measures of physical activity can be reported.

By examining studies aimed at considering the relationships between barriers and level of physical activity, the majority of them reported lack of time, lack of interest, fear of injury or bad health conditions as barriers to regular physical activity for sedentary people [53,54]. In contrast, for active older people, the results are less obvious. Two studies out of 21 reported no distinction between active and nonactive older people in terms of barriers [42,55]. Four studies reported a lack of time as a barrier for active older people [56,57,58,59], and two studies reported weather [56,57] and health problems [57,58] as barriers. The other studies only reported that scores of barriers are significantly higher for sedentary older people than for active older people. In contrast, it appears that the number of barriers evoked is a good index of level of activity, in that older people who perceived fewer barriers were more likely to engage in moderate or greater intensity activity [60,61]. In a similar vein, another study showed that older adults in the action and maintenance stages have high levels of physical activity and perceive fewer barriers to being active [62]. Interestingly, these results are not sufficient to determine, for instance, the weight of a barrier on the likelihood of relapse in active people. These inconsistent results have led some authors to suggest that perceived barriers could have a significant impact only on those who had lower levels of exercise but almost no effect on those who were regularly engaged in exercise [43,48]. However, 50% of active people drop out before making physical activity a habit. Details of the major studies that have examined the relationship between barriers and exercise behavior are presented in Table 1.

To sum up, this section highlights two interesting conflicting findings. First, some studies have shown that regardless of the level of engagement in physical activity, the barriers remain the same. However, older people who are active or have already been active will be more sensitive to environmental (e.g., weather) barriers than people who never have been active. This point deserves further examination. Second, the number of obstacles to participation in physical activity requires further studies, as a single barrier may be enough to disengage someone from physical activity (e.g., lack of time or health issues). No need to accumulate barriers to stay inactive.

### 4.2. Relationship of Barriers with Exercise-Related Stages of Change (N = 4)

In this section, two concerns are addressed: (1) measures of perceived barriers and stages of change and (2) barriers cited in the pre-action and post-action stages. The measures of barriers suggested by the TTM are applied in three out of five studies [69,70,71]. However, formulations are not always in accordance with perceived barrier definitions in TTM [72]. It is necessary to define whether the barriers prevent exercise or whether the barriers are negative consequences of exercise. This distinction is important because it leads to the identification of two different determinants of nonadherence. Moreover, the barriers concern different intensities of physical activity, such as barriers to moderate activity, barriers to strength training or barriers to insufficiently active people [54,69,70]. Consequently, one can assume that the barriers differed depending on the intensity of exercise training. By examining the statements of the five stages of change, some interesting differences appeared. One stage out of five has the same formulation across studies and concerns the precontemplation stage (i.e., “No, I do not exercise regularly” or “I do not intend to in the next 6 months”). In contrast, contemplation and preparation stages are differently formulated, which can weaken the results. More particularly, two different formulations can be reported for the contemplation stage through intention (i.e., “Not exercising but intended to”) and attitude (i.e., “Did not engage but were thinking of” or “Considering participating”). Concerning the preparation stage, people are considered to be active but not regularly [69,72] or not active but intend to or plan to [70,73]. For the action stage, a consensual formulation implies regular exercise but for less than 6 months. Finally, for the maintenance stage, older adults have been exercising regularly for more than 6 months.

In the same manner as in the previous section, barriers are relatively well identified with sedentary people (i.e., precontemplation and contemplation stages) but not in later stages. For older people in precontemplation and contemplation stages, a lack of time (too busy), being too tired and bad health are most often cited as barriers [71,72]. However, these three barriers are generally evoked regardless of the stages of change. A lack of motivation (laziness) appears in pre-action stages (including PC, C and PR). From the time when people consider physical activity until the beginning of physical activity, caregiving and a lack of energy may hinder their intention to become physically active. In the action stage, two other barriers can be cited: a fear of injury and self-consciousness about one’s own appearance [72]. One study examined specific barriers for older people in action or maintenance stages [71] and identified health problems and time constraints for older people who quit exercise. Overall, too few studies have examined barriers in action and maintenance stages, and it could be interesting to examine the weight of environmental barriers on these two later stages. Details of the four major studies that have examined the relationship between barriers and exercise-related stages of change are summarized in Table 2.

In summary, the first two sections that discussed the relationship between barriers and exercise behavior and stages of change show that while there are links that are established, the results are not generalizable due to several methodological differences. These differences include the use of different definitions of the perception of the barrier when measuring it in different studies, the use of different tools to measure physical activity levels and the lack of consensus in the preparation stage.

### 4.3. Relationship of Barriers with Exercise Behavior and Stages of Change

Eight studies were identified that examined the relationship between barriers, exercise behavior and exercise-related stage of change. Five of the eight studies are based in part on TTM, and three are not theory-based. The measurement of perceived barriers is somewhat confusing going from unidimensional measure [25] to a score taking into account the number of barriers [74] via subscales assessing barriers [26,75] or decisional balance scale [76]. Stages of change were measured either by the number of prescribed exercises on the number of exercises reported or by the use of stages of change questionnaires. Here, again, confusion appears in the formulation of stages, particularly in contemplation and preparation stages. The level of physical activity was measured differently through light physical activity, leisure time physical activity, physical activity prescription schemes, physical activity daily life or exercise logs. Among the main results, it appears that barriers are correlated with exercise behavior [25,26,75,77] and stages of change [26,27,66]. However, inconsistent with the TTM, three studies reported that the stages of change are not a mediator between barriers and exercise behavior in older adults [25,74,76]. Moreover, three studies reported that barriers are not predictors of exercise behavior [25,75,76].

## 5. Discussion

Do these findings question the relevance of the transtheoretical model among a healthy elderly population? Before questioning the consistency of the model with older healthy people, let us return for a moment to the results. Why is the hypothesis difficult to explore? Based on this review, four explanations can be reported: the lack of a clear definition of each stage of change and, more specifically, the contemplation and preparation stage; the definition and formulation of perceived barriers; the measurement of physical activity; and the distinction between barriers that hinder the transition between each stage of change from those that hinder the increase in the level of physical activity.

### 5.1. Definition and Formulation of Perceived Barriers

The definitions of perceived barriers make the examination of the results complex for the two following reasons. First, the definition does not always fit with the theoretical model. Second, the formulation of the items is not always in concordance with the definition. For instance, in King and colleagues’ study, the authors asked older people to rate whether the following conditions (i.e., barriers) were present in the participant’s neighborhood [78]. This question does not provide information on the real barriers faced by people. Other questionnaires asked why people are not engaged in regular physical activity, or what limits or inhibits their exercise behavior. The response provides information on potential barriers but not on the frequency or the weight of the barriers. Finally, some authors assessed the degree to which certain factors prevent people from exercising. In accordance with the definition of perceived barriers in the TTM literature, barriers represent the negative consequences of the target behavior. For instance, the following formulation was proposed by Budnick et al. and was in line with the TTM-based conceptualization of barriers: “What do you think will be the consequences if you exercise regularly? I will not have enough time to care for my relative anymore” [70]. By contrast, when authors questioned “How often do the following things prevent you from exercising?”, the question is closer to barriers defined by SCT. Here, perceived barriers are defined as barriers that prevent individuals from initiating a health behavior such as exercising. Barriers provide information about the potential constraints on action as perceived by individuals. Generally, this kind of barrier is reported more specifically by vulnerable persons, such as cancer survivors, for whom experienced nausea, fatigue, a lack of time and a lack of external support contribute to directly hindering exercise as a routine [79,80,81,82]. This distinction is not obvious in studies, and authors have to appropriately formulate perceived barriers when using the TTM as a theoretical framework. To accurately assess perceived barriers relative to decisional balance, authors have to ask participants about barriers that represent negative consequences of the practice itself, such as fatigue and pain. By contrast, when authors are interested in the measure of barriers relative to SCT, they have to ask about barriers that prevent people from exercising. Therefore, future studies should differentiate between barriers that are obstacles to engaging in a behavior and costs that are the negative consequences of engaging in a behavior. In looking at how barriers were measured in the different studies, it was found that several tools are used, namely, questionnaires and interviews. Moreover, questionnaires and interviews do not have the same content. It would, therefore, be interesting for researchers in the field to work in synergy for the co-construction of a standard tool to measure barriers to the practice of physical activity in older adults.

### 5.2. Methodological Issues

#### 5.2.1. Conceptualization of Stage of Change

Among the eight studies [25,27,69,70,71,72,73,75] assessing stages of change, the formulations used for precontemplation, action and maintenance stages were consistent with the TTM. Indeed, PC concerns individuals who do not exercise regularly and who do not intend to exercise in the next 6 months. Likewise, action and maintenance stages were formulated in the same manner whatever the study, i.e., individuals who exercise regularly but for less than 6 months for the former and more than 6 months for the latter. In contrast, the formulations of contemplation and preparation stages are associated with intention to change or thinking about change. According to Marcus and Forsyth, the contemplation stage is referred to as thinking about change but thinking about it cannot be considered an intention [35]. Concerning the preparation stage, for some authors, people in this stage are active, whereas for other authors, people in this stage are not active. For Marcus and Forsyth [35], the preparation stage concerns active persons but not at levels meeting the CDC/ACSM guidelines [36]. For other authors, preparation is the stage of decision making, whereas in the contemplation stage, individuals are aware that they have a problem and are seriously thinking about resolving it, but they have not yet made a commitment to take action in the near future [83]. Concerning the measure of stages of change, some guidelines must be reminded. Based on Marcus and Forsyth’s recommendation [35], stage 2 or contemplation stage refers to inactive and not thinking about becoming more active. Nevertheless, thinking about exercise is not an intention, and when authors formulated this stage by using “intend to” or “think of”, the impact on exercise behavior should be weaker for the latter. The same remark can be made concerning the preparation stage in that depending on the degree of commitment in exercise (from not exercising to engage irregularly), the stage does not affect exercise behavior in the same manner. According to Marcus and Forsyth [35], stage 3 refers to performing some physical activity but not at levels meeting the CDC/ACSM guidelines [36]. Based on these observations, future studies must first harmonize their definitions of contemplation and preparation stages with the definitions in the TTM. Particularly regarding the stage of preparation, we suggest two proposals for this harmonization. A first proposal would be that future studies measure the stage of preparation by focusing only on the fact that the participant has started the practice but has not yet reached the required level. A second proposal would be to subdivide the preparation stage into two substages. A first substage would be intentional preparation, where participants identify their inactivity and plan to begin practice in the very near future. The second substage would be active preparation, where participants are active but insufficient. In short, standardizing the stages of change, particularly the stages of contemplation and preparation, would make it possible to harmonize the results.

#### 5.2.2. Limits to the Measurement of Physical Activity

One of the findings of our narrative review is that almost all of the studies used self-reported questionnaires to measure physical activity levels. However, the literature shows that self-reported questionnaires have three limitations [38]. The first limitation is that self-reported questionnaires do not measure the proportions of aerobic and resistance activities or the environmental context. The second limitation concerns the high variability of the period surveyed and information collected, which exists between self-reported questionnaires. Finally, the third limitation is that even if the questionnaires are valid, their reliability is highly dependent on the length of the activity recall period and the respondent’s level of physical activity. To address the uncertainties and subjectivity that may be present in self-reported questionnaires, some studies have used accelerometers. Indeed, recent studies have shown that the use of accelerometers in older adults to measure physical activity levels provides more reliable information [84,85]. However, it is difficult to use accelerometers over a long period of time [86]. In addition, accelerometers underestimate many activities, such as cycling, swimming and resistance training [87]. A second important finding is that when we compare the level of physical activity identified in the studies to the classification of Sedentary Behavior Research Network (SBRN) [88], several problems arise. For instance, depending on the study, the level of physical activity was measured over a month, a week, a day, a typical week over the past month or the past 2 weeks. Likewise, the frequency and duration and total time engaged in physical activity were measured nine times for both intensity (three times). The diversity of measures complicates the comparison of results. In addition, the strength of the relationships observed between the barriers and the level of activity depends on the type of measurement. To avoid the loss of information due to long- or short-term recalls, it has been suggested to keep the reporting interval relatively short (no longer than three months) and that when the recording period is less than one week, both weekday and weekend activities must be included [38]. Based on this suggestion, future studies should standardize the measurement of physical activity levels before relating them to barriers and stages of change. We propose that the measurement of the physical activity level be carried out by coupling self-reported questionnaires (subjective assessment) and an accelerometer (objective assessment). The self-reported questionnaires would be used for the last three months, and the accelerometer (ActiGraph GT9X Link) would be used for one week. The benefit of using the accelerometer would be to cross-reference the latter’s data with the data from the self-report questionnaires. However, this is also to counteract the social desirability bias that can be observed when using non-validated self-report questionnaires.

### 5.3. Identification of Barriers That Predict Behavior Change

It appears that some barriers are the same when older people are sedentary, that is, in pre-action stages of change (PC and C) or at levels of activity under 1.5 kcal·day^−1^·kg^−1^. These barriers are, in order of importance, poor health, lack of time, lack of motivation, tiredness and environmental barriers (e.g., lack of safe place, weather). In contrast, the results are less obvious when people are either insufficiently or sufficiently active. A lack of time is the most commonly reported barrier in studies [57,59,63,64,71,72]. Two studies reported self-consciousness about physical appearance [65,72]. Except for a lack of time, no other barriers appeared in these later stages of change, and this review highlights that barriers that hinder stages of change are not exactly the same as those that hinder levels of physical activity. Environmental barriers are more likely to hinder exercise behaviors in active people. When people are active, they better self-regulate their behavior and are more able to overcome personal barriers. Moreover, very few studies have examined the reasons for relapse in terms of barriers for individuals in action or maintenance stages. It would seem that readiness to change (or intention) is a mediator in early stages but that this mediator effect disappeared in later stages of change when individuals are active. Presumably, if barriers prevent engagement in physical activity, then they will certainly strongly predict the intention as well as the level of activity of people until they reach the action stage. By contrast, if barriers are defined as the unpleasant consequences of the targeted behavior, according Gallagher et al., they can play a more important role in the action and maintenance stages [89].

In brief, TTM studies are generally based on social-cognitive theory, which considers health behavior change as a continuous process and assumes that the theory’s factors are relevant for all individuals. As suggested by Schüz et al. [90], the idea of qualitative differences between stages implies that people face different variables that determine these transitions. If these qualitative differences were confirmed, behavior-change interventions could be tailored to stages to support individuals in mastering stage-specific barriers and moving towards the next stage. Consequently, the use of a unidimensional scale cannot help to understand the relationships.

### 5.4. Limits

A first limit of our work was that four of the studies [27,41,45,72] presented in our narrative review involve a part of participants between the ages of 18 and 49. However, the results of these studies are not substantively different from those that have included only older adults. Another limit was search terms are rather limited. Indeed, we did not include “Ageing” in our keywords because previous systematic and integrative reviews have not used this keyword [9,14,15,28,29]. However, we conducted an extensive literature search of several databases to identify as many publications as possible in order to include the most relevant ones. Thus, the omission of a few papers due to the non-use of the keyword “Ageing”, would not have a significant impact on the results obtained in this review.

## 6. Conclusions

This review highlights the barriers reported by older people in different stages of behavior change. Helping people overcome barriers to physical activity is an intervention that successfully guides physical activity programs, but identifying these barriers before engaging in physical activity could help avoid adherence failures and attrition. In addition, this work shows that barriers correlate with exercise behavior and stages of change in the majority of studies that have been reviewed on this topic but rarely predict exercise behavior change. However, future studies need to consider standardizing the items used to measure the stages of change and the definition of barriers. It also seems essential that in future studies, diversification of the measurement of physical activity should be avoided. From this perspective, it would be interesting to standardize the measurement of physical activity levels by completing subjective measures with objective measures in future research programs aimed at clearly establishing the relationships between barriers, stages of change and exercise behaviors.

## Figures and Tables

**Table 1:** Summary of studies that have examined the relationship between barriers and exercise behavior.

Reference		Participants and Demographics									
		Aims Related to the Study	Theoretical Framework	Total Participants and Gender	Age Range	Measure of Barriers	Outcomes Variables	Results			
								Sedentary (or Nonexercisers)	Insufficiently Active (or Light Walking)	Sufficiently Active (or Moderate and Vigorous)	Quitter
								<1.5 kcal·day^−1^·kg^−1^	≥1.5-<3 kcal·day^−1^·kg^−1^	≥3 kcal·day^−1^·kg^−1^	
[41]	Bautista et al. (2011)	To examine barriers to exercise among Hispanics	-	398—Men and women	18–96 years old. >50 years = not available	Constructed by the authors-Total number of barriers (frequency)	Level of engagement in exercise in the past 30 days	Lack of time, too tired, lack of self-discipline	-	-	-
[63]	Biedenweg et al. (2014)	To explore barriers to physical activity program participation	-	39—Men and women	Not reported (in their early 70s)	Generated by interviews	Programs supporting exercise behavior	Already achieve sufficient exercise, lack of motivation, not ready, poor health	-	-	Not having enough time, lack of affiliation with people in the program
[42]	Bird et al. (2009)	To determine the factors associated with physical activity participation (based on an ecological model)	-	72—women	60–84 years old	The St Louis Scale (Brownson et al., 2004) + NEWS	IPAQ	No barriers are predominantly cited by these three groups			-
[43]	Booth et al. (1997)	To examine the barriers to regular participation in physical activity reported by insufficiently active older adults	-	449—Men and women	60 years and older	The authors proposed 19 barriers based on earlier Australian study (Owen and Bauman, 1992)	Leisure-time physical activity during the previous 2 weeks	Injury, poor health, too old		-	-
[53]	Booth et al. (2000)	To identify perceived environmental influences associated with physical activity participation	TPB	449—Men and women	60 years and older	Constructed by the authors-Physical environments	Leisure-time physical activity during the previous 2 weeks	Poor health, lack of local hall, lack of recreation center, lack of safe footpath, risk of harm	-	-	-
[64]	Brittain et al. (2012)	To examine in barrier limitation between sufficiently and insufficiently active participants	SCT	109—Women	50–75 years old	Barriers (Gyurcsik et al., 2009) + barrier limitations	IPAQ	Non-work-related priorities, work, family obligations, lack of energy/fatigue		Non-work-related priorities, work, family obligations	-
[44]	Cohen-Mansfield et al. (2003)	To ascertain perceived barriers to exercise	-	324—Men and women	74–85 years old	Open-ended approach	PASE + Exercise leisure time during the preceding 7 days (Washbum et al., 1999).	Bad health, lack of motivation, dislike exercise	-	-	-
[61]	Dawson et al. (2007)	To investigate whether low levels of walking were associated with health problems and environmental	HBM, SCT and ecological model	680—Men and women	50 years and older	Barriers to walking around their neighborhood (Booth et al., 2000)	Amount of physical activity during the preceding 7 days-British heart foundation’s daily activities questionnaire (Taylor et al., 1978)	Health problem, more than one environmental barrier		-	-
[54]	Moschny et al. (2011)	To analyze barriers to physical activity	-	1937—Men and women	72–93 years old	Frequently reported barriers	Determined from the barrier questionnaire: participants were asked about their physical activity	Poor health, lack of company, not interested		-	-
[12]	Horne and Tierney (2013)	To explore the barriers to initiating and maintaining regular physical activity	-	116—Men and women	60–70 years old	In-depth interview	Department of Health guidelines (2009)	Poor health, loss of self-confidence, lack of belief in their own physical ability, fear of increasing their symptoms, lack of knowledge		-	-
[45]	Juarbe et al. (2002)	To examine the factors that influence women’s ability to engage in exercise behavior	-	143—Women	40–79 years old > 50 years = 89	One open-ended questions on barriers encountered in staying physically active	7-Day Physical activity recall (Mayer et al., 1991)	Time constraints and women’s roles, personal health, internal (lack of determination, lack of motivation) and external factors (lack of facilities, weather, lack of safe place)		-	-
[65]	King et al. (2000)	To explore personal and environmental barriers to physical activity	Ecological model	1791—Women	50 years and older	10 frequently reported personal barriers to physical activity (Dishman and Sallis, 1994) + environmental barriers	Leisure-time physical activity, occupational activity and physical activity occurring around the home	Too tired, bad health, lack of energy, presence of unattended dogs	-	Self-consciousness about physical appearance	-
[66]	Kleppinger et al. (2003)	To determine if health perception could identify people more likely to adhere to exercise	-	189—Women	59–78 years old	SF-36 (Ware and Sherbourne, 1992)	Exercise logs			Vitality, role-emotional, bodily pain, social functioning, and mental health	Problems with role-emotional and social function
[67]	Kowal and Fortier (2007)	To examine relationships between barriers and environmental characteristics and physical activity behavior	Ecological model	90—Women	50–68 years old	The reasons for not engaging in more physical activity over the past 6 months + environmental characteristics	List of physical activities (i.e., leisure-time activities, sports, and home-based activities)	Laziness, too tired, lack of interest, daily activities	Cost (Progressed group but not stable active)	-	Too tired (Regressed group)
[57]	Lees et al. (2005)	To determine barriers to the exercise behavior	-	66—Men and women	65 years and older	Open-ended approach	Planned physical activity (3 times/week during at least 20 min)	Fear of injury, inertia, negative affect, weather, inconvenience, time discomfort, perceived capability, physical ailments, too old	-	Inertia, lack of time, physical ailments, inconvenience, perceived capability, discomfort, verbal persuasion, too old, weather, negative affect	-
[58]	Lim and Taylor (2005)	To examine factors associated with physical activity	-	8881—Men and women	65 years and older	Open-ended approach	“Adequate” physical activity was defined as at least 30 min of walking, moderate or vigorous activity on at least 5 days in the last week.	Health problem, too busy, pain		-	
[26]	Newson and Kemps (2007)	To identify factors that prevent from exercising and to examine how they relate to intentions to exercise	-	217—Men and women	63–86 years	Barriers exercise based on Larkin (2005)	PARS (Jackson et al., 1990)	Medical, concerns (pain, lack of energy) facilities and knowledges			
[46]	Reichert et al. (2007)	To examine the association between barriers and participation in leisure-time physical activity	-	358—Men and women	65 years and older	8 perceived personal barriers	IPAQ	Lack of time, dislike exercise, too tired, lack of company, lack of money	-	-	-
[59]	Salmon et al. (2003)	To examine the associations of physical activity and sedentary behavior with barriers	BCT	308—Men and women	60 years and over	Constructed by the authors-Environmental and personal barriers	1-week leisure-time physical activity recall measure + leisure-time sedentary behavior	Lack of time, family commitments, feeling tired, pollution, cost	Work commitments	Other priorities, work commitments, cost	-
[55]	Smith et al. (2012)	To identify the relationship between what older adults perceived as barriers to physical activity and participation	-	4900—Men and women	60 years and older	Based on CCHS-HA, 13 barriers have been proposed	PASE	Activity not available (for males), lack of time (for females)	No barriers	-	-
[68]	Wilcox et al. (2000)	To examine the pattern of relations between barriers and LTPA	-	1422—Women	50 years and older	Perceived barriers to LTPA + Environmental characteristics	LTPA during the past two weeks	Lack of enjoyable scenery, not frequently seeing others exercise, greater barriers, less social support (rural women), too old, greater barriers, less social support (urban women)	-	-	-

BCT = Behavioral Choice Theory; CCHS-HA = Canadian Community Health Survey-Healthy Aging; ERA-38 subscales = Expectations Regarding Aging; HBM = Health Behavior Model; HPM = Health Promotion Model; IPAQ = International Physical Activity Questionnaire; LTPA = Leisure Time Physical Activity; NEWS = Neighborhood Environment Walkability Scale; PASE = Physical Activity Scale for the Elderly; PARS = Physical Activity Rating Scale; SCT = Social Cognitive Theory; SF-36 = Short-Form Health Survey; TPB = Theory of planned Behavior.

**Table 2:** Summary of studies that have examined the relationship between barriers and exercise-related stages of change.

Reference		Participants and Demographics											
		Aims Related to the Study	Theoretical Framework	Total Participants and Gender	Age Range	Measure of Barriers	Outcomes Variables	Results					
[72]	Heesch et al. (2000)	To identify the barriers most likely to interfere with exercise participation at each stage	TTM	2912—Men and women	40 years and older. >50 years = 1791	Adapted from the San Diego Health and Exercise Survey	Five-item instrument developed by Marcus, Rossi et al. (1992)	**PC**	**C**	**PR**	**Act**	**M**	**R**
								Too tired, lack of energy, bad health, lack of time	Lack of time, too tired, caregiving, lack of energy, lack of safe place	Lack of time, self-consciousness of appearance, too tired, lack of energy, fear of injury, caregiving		-	-
[27]	Sorensen and Gill (2008)	To examine relationship between the experienced barriers and exercise-related stages of change	TTM	4921—Men and woman.	30–75 years. > 50 years = 1850	Constructed by the authors and based on Brawley et al. (1998)		Health barriers (60–75 years men and women) > M: Practical barriers (75-year-old men) PR-C > M: Affective/Cognitive barriers (60-year-old men)	Health barriers (60–75 years women) > M: Practical barriers (60-year-oldmen) > M: Affective/Cognitive barriers (60-year-old women)	> M: Priority barriers (60-year-old men)			
[73]	Thorgensen-Ntounami (2009)	To examine the usefulness of an ecological model in predicting Soc	TTM	318—Men and women	61–81 years	Environmental conditions in their neighborhood	Stages of change using the five-item stages of change short form scale			Heavy traffic and presence of unattended dogs	-	-	-
[71]	Walcott-McQuigg and Prohaska (2001)	To examine factors influencing exercise behavior	TTM	103—Men and women	55 years and older	Generated by interviews	Motivational readiness for exercise (Marcus et al., 1992)	Health problems, PA makes tired, laziness, time constraints (female) Exercise from housework		Health problems, time constraints (female)			-

Note: PC = Precontemplation; C = Contemplation; PR = Preparation; Act = Action; M = Maintenance; R = Relapse; TTM = Transtheoretical Model of change.
